# Abnormal ERV Expression and Its Clinical Relevance in Colon Cancer

**DOI:** 10.3390/genes16080988

**Published:** 2025-08-21

**Authors:** Aditya Bhagwate, William Taylor, John Kisiel, Zhifu Sun

**Affiliations:** 1 Division of Computational Biology, Mayo Clinic, Rochester, MN 55905, USA; bhagwate.adityavijay@mayo.edu; 2 Division of Gastroenterology and Hepatology, Mayo Clinic, Rochester, MN 55905, USA; wtaylor@mayo.edu (W.T.); kisiel.john@mayo.edu (J.K.)

**Keywords:** human endogenous retrovirus, ERV, colon cancer, tumor immune response, patient survival

## Abstract

Background/Objectives: Human endogenous retroviruses (ERVs) are genomic sequences integrated into the human genome from ancestral exogenous retroviruses and are epigenetically silenced under normal conditions. Growing evidence has shown that they can be reactivated in human diseases such as cancers and autoimmune diseases. However, their clinical implications in colon cancer are yet to be explored. Methods: RNA-seq data were downloaded from RNA Atlas and TCGA for cell lines and tissue samples, respectively. After alignment, ERV expression was quantified against comprehensively compiled ERVs (3220). ERV expression profiles were compared between sequencing protocols, cancer and normal cells, and matched tumor and normal tissue pairs. Unsupervised clustering was used to identify ERV-defined tumor subtypes and their associations with clinical and other molecular features. ERV association with disease-specific survival (DSS) was performed using the Cox regression model. Results: PolyA and total RNA protocols were comparable in ERV expression detection. Cancer cells had significantly increased ERV expression and reactivation. Upregulated ERVs were significantly enriched in viral protein interactions with cytokine and cytokine receptors. ERV expression-defined tumor classes were significantly associated with tumor mutation burden and immuno-phenotypes such as antigen processing and presenting machinery and tumor immune infiltration score. Survival analysis identified 152 ERVs to be independently associated with DSS. Conclusions: ERV abnormal expression is common in colon cancer. The ERV-defined subtypes are associated with tumor immunity, and some ERVs are independently associated with patient outcomes.

## 1. Introduction

Human endogenous retroviruses (HERVs or ERVs) are genomic sequences integrated from ancestral exogenous retroviruses. They account for nearly 9% of human DNA [1]. ERVs are mostly expressed during embryogenesis but are believed to be epigenetically silenced afterwards [2]. Recent studies have shown that certain ERVs may be re-activated for transcription in certain diseases such as cancers [3,4,5] and autoimmune diseases [6]. These activated ERVs may trigger an immune response and can have treatment implications. Indeed, studies have indicated that ERV DNA can be transcribed to double-stranded RNA that is sensed by immune system as a “danger signal”, leading to a viral mimicry state [3]. The viral DNA can also be transcribed to mRNA which is then translated into proteins with foreign tumor antigens [7]. The expression of certain ERVs was shown to be associated with immunotherapy response [8].

Most studies investigated selected or a small subset of ERVs [4,8], although some recent studies took an unbiased approach for all possible ERVs based on their sequence features (interspersed repeats and low complexity) using RepeatMasker (https://www.repeatmasker.org/), where over 700K ERVs were analyzed [3]. The vast majority of studies so far have used RNA-seq data generated by polyA enrichment protocol. One of the often asked questions is whether ERV transcripts have a polyA tail and whether ERVs detected from this protocol are representative.

In this study, we performed comprehensive profiling of well annotated 3220 ERVs in colon cancer. We started with profiling ERV expression in cancer and normal cells with both PolyA and total RNA protocols to evaluate the completeness of ERV detection from the commonly used polyA RNA-seq data. We then compared the expression patterns between tumors and their adjacent normal tissues. Unsupervised clustering was used to identify ERV expression-defined subtypes and their association with other phenotypes. ERV association with patient survival was also explored for potential prognostic markers.

## 2. Materials and Methods

### 2.1. Manually Compiled ERV Set

We used a previously compiled list of 3220 ERVs as described by Tokuyama [6]. These ERVs were either transcribed in various disease contexts or identified as ERVs based on sequence analysis in silico. These collections were enriched in autonomous RTR elements with an average length of 7 kb and were found to have cell-type-specific expression patterns, and some were significantly increased in the peripheral blood mononuclear cells of patients with systemic lupus erythematosus [6].

### 2.2. RNA-Seq Data

To evaluate the potential impact of RNA-seq library preparation protocols on ERV expression, we downloaded seven colorectal cancer (CRC) samples, one normal colon epithelium sample, and one monocyte sample from GEO (GSE138734) as described in the RNA catalog project [9]. All samples except the normal colon have both polyA and total RNA ribosome removal library preparation generated data while the normal colon cell sample only has polyA prepared data.

The main RNA sequencing data, which include 307 primary tumors and 41 adjacent normal tissues (25 are paired from the same patients) from patients with colon adenocarcinoma (COAD), were downloaded from GDC TCGA (https://portal.gdc.cancer.gov/, accessed on 22 June 2023) and dbGAP (https://www.ncbi.nlm.nih.gov/gap/, accessed on 20 July 2023).

### 2.3. RNA-Seq Data Pre-Processing and Analysis

The downloaded raw RNA sequencing data were first converted into fastq and then processed using our MAPR-Seq pipeline [10] with STAR aligner. ERV expression was quantified against the comprehensively compiled ERV catalog as described above where only uniquely mapped and non-junction reads into the coordinates were counted (i.e., reads that span the region which are likely from another gene due to splicing were excluded).

ERV expression in cell lines from both polyA and total RNA preparations were compared for the same biological sample for their correlation and the number of ERVs detectable from each.

ERV expression profiles and differential expression were first performed between matched tumor and normal pairs (from 25 patients) with edgeR (paired design). The overlapping or closest protein coding genes for the differentially expressed ERVs were used for pathway enrichment analyses, separately for up- and down-expressed ERVs. The DE ERVs were also compared with the analysis using all tumors and normal samples for consistency with edgeR (unpaired design).

### 2.4. ERV-Defined Tumor Subtypes and Their Association with Clinical and Other Molecular Phenotypes

Unsupervised clustering was used to identify ERV expression-defined tumor “subtypes”, and their associations with clinical and other molecular features were explored. The comprehensive clinical and molecular profile data was obtained from the integrated clinical data resource [11] while immunogenic profiles such as antigen processing and presenting machinery (APM), immune infiltration score (IIS), and tumor mutation burden (TMB) were obtained from a previous publication [12]. Higher scores of these immunogenetic parameters indicate a tumor is “hot” in the immune response and more likely to respond to immune checkpoint inhibitor treatment. The association between ERV-defined tumor subtypes and these continuous variables was tested by ANOVA. The disease specific survival (DSS) was performed using the Kaplan–Meier model. A *p*-value less than 0.05 was considered as being indicative of significant association.

### 2.5. ERV Expression Association with Patient Survival

ERV association (normalized log2 RPKM expression) with DSS was performed using the Cox multi-variable regression model (on 285 unique patients) with patient age, sex, and tumor stage as covariates, where an association *p* value of less than 0.05 for an ERV was considered to be indicative of significant association. Multiple testing was corrected using the q-value implemented in the R package “qvalue”. Kaplan–Meier curves were used to visualize selected ERV association by binarizing ERV expression into high or low expression based on the median expression with a log rank test.

All analyses were performed using R (v4.2.2, https://www.r-project.org/) and relevant Bioconductor packages as mentioned above (http://bioconductor.org/).

## 3. Results

### 3.1. Comparable ERV Expression Measurement in CRC Cell Lines Between Total RNA rRNA Depleted- and Poly a-Enriched RNA-Seq Data

**Figure 1:**
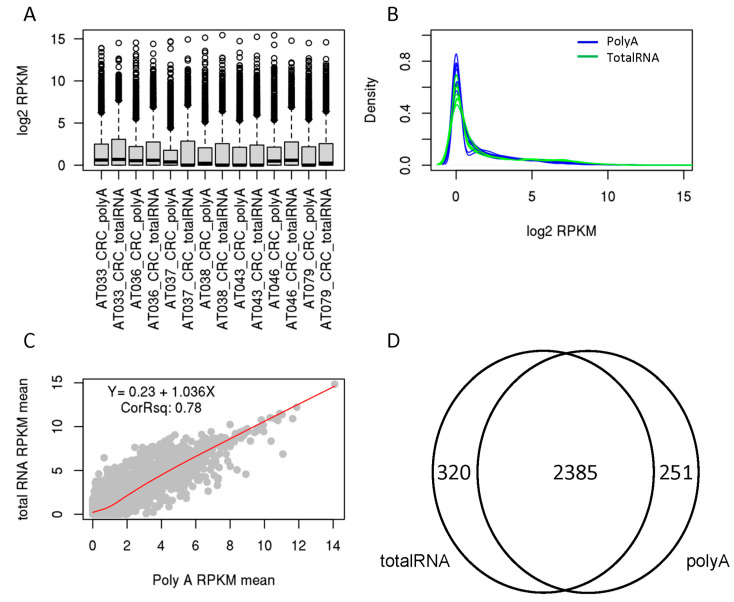
ERV expression in CRC cell lines from both PolyA and total RNA protocols. (**A**) For each pair of PolyA and total RNA samples, ERV expression was slightly higher in the total RNA sample as measured by the median (horizontal bar within the box) and 75% percentile log2 expression (upper end of the box). The dots above the box are ERVs with very high expression (>75% percentile). (**B**) The density plot shows the PolyA samples have more ERVs expressed at lower level than the total RNA samples. (**C**) The correlation plot of the means from both protocols shows good correlation, although total RNA data has more ERVs above the diagonal line (and a positive correlation coefficient). Each dot represents an ERV. The dots above the red line were higher in total RNA protocol. (**D**) Venn diagram of common and unique ERVs detected by two different protocols.

To address the question of whether polyA-enriched RNA-seq is adequate or suitable for ERV analysis, we first performed analysis on seven CRC cell line data from the RNA Atlas project, where both polyA and total RNA library preparation protocols were applied [9]. Among 3220 ERVs, 2956 had non-zero expression across seven cell lines in either polyA or total RNA protocol. The numbers of ERVs detected in the two protocols were quite similar. While the mean number in polyA protocol samples was 1560 (median 1565), it was 1560 (with median of 1485) in the total RNA samples. Comparing two libraries for each pair of samples showed that ERVs from total RNA protocol had slightly higher overall expression (as shown in the box and density plot, Figure 1A,B). The correlation coefficient R square between the mean expression of seven replicates between two libraries was 0.78 (Y = 0.23 + 1.036X, R = 0.88, Figure 1C; pair-wise correlation for each cell line is provided in Figure S1). For the ERVs that were detected in both polyA and total RNA protocols, 1803 were higher and 1153 were lower in the total RNA data. There were 320 and 251 ERVs detected exclusively in the total RNA and polyA protocol, respectively (Figure 1D). These data provide evidence that although there are protocol differences, the most commonly used polyA protocol can be used to profile the majority of ERVs, which is important as the vast majority of the public RNA-seq repositories are generated using this protocol, for example, the commonly used The Cancer Genome Atlas (TCGA) project and GTex project (https://gtexportal.org/home/). Total RNA protocol is still preferred though, as it detects slightly more ERVs.

### 3.2. Low Number and Expression of ERVs in Normal Cells Compared to CRC Cells

**Figure 2:**
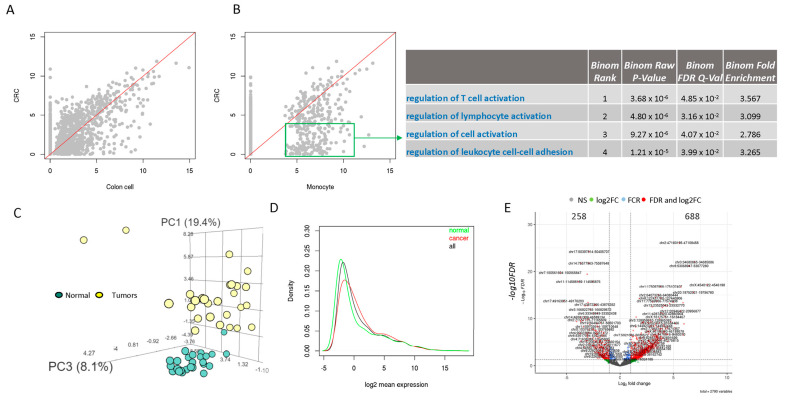
ERV expression in CRC cells, normal cells, primary CRC tumors, and adjacent normal colon tissues (**A**) In CRC vs. normal colon cells, many ERVs are not expressed in normal cells while the majority are higher for those commonly expressed. (**B**) CRC cells vs. monocytes. Many ERVs are expressed in CRC cells but not in monocytes. For those commonly expressed, many are higher in monocytes, which are enriched in immune-related pathways. (**C**) PCA shows tumors are clearly separated from normal samples. (**D**) More ERVs are expressed more highly in tumor samples from the density plot (red line shifted to the right). (**E**) Many ERVs are differentially expressed, with more than 688 upregulated in the tumors (vs. 258 downregulated).

It is believed that ERVs are suppressed in normal human cells [2]. To confirm if this is true in more pure cell lines, we looked at the ERV expression in normal colon cell and blood monocytes from the RNA Atlas project. Among the 3220 ERVs, the colon and monocyte cells had 921 and 312 ERVs with detectable expression, respectively (at least one mapped read), which is dramatically lower than in the CRC cells (ranging from 1124 to 1895). Many ERVs were only expressed in CRC cells, while for those in common, most of them were expressed higher in CRC cells (Figure 2A). ERV expression was also cell-type specific. In comparing CRC cells with monocytes, many ERVs were found to be only expressed in CRC cells, while others were expressed less relative to the monocytes (Figure 2B). The ERVs that were more highly expressed in monocytes were enriched in several immune cell regulation processes (Figure 2B), although no enrichment was observed for the ERVs that were highly expressed in CRC cells, likely because they are from non-coding regions of the genome getting re-activated in cancer, and the lack of pre-defined pathways. To confirm if this is a universal phenomena in cancer, we profiled three more cancer cell lines with matching normal cells including breast, lung, and prostate cancer, and found that all cancer cell lines have a higher number of ERVs that are detectable and have a higher level of ERV expression (Figure S2), supporting the assertion that EVRs are reactivated in cancer.

### 3.3. Most ERVs Are Overexpressed in Primary Tumor Tissue Samples Compared to Their Paired Normal Colon Samples

**Figure 3:**
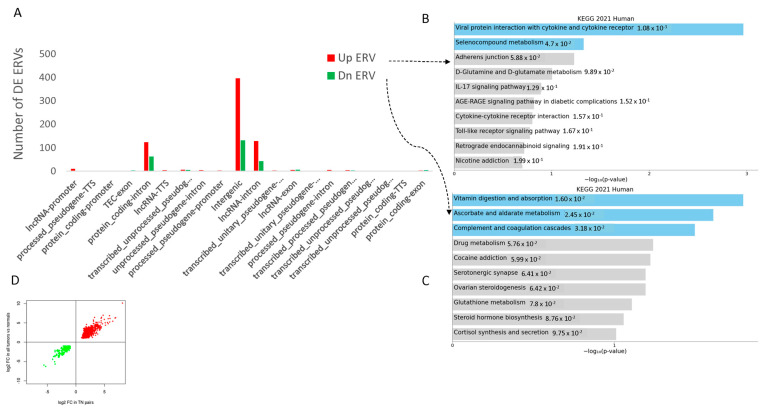
The distribution of DE ERVs in genomic locations and their pathway enrichment. (**A**) DE ERVs in different locations of the genome, separately for those higher or lower in tumor samples. (**B**) Pathway enrichment for the higher ERVs using their overlapping or nearby annotated genes. Pathways highlighted with blue are significantly enriched (*p* value less than 0.05) while those with grey are not. (**C**) Pathway enrichment for lower ERVs using their overlapping or nearby annotated genes. Pathways highlighted with blue are significantly enriched (*p* value less than 0.05) while those with grey are not. (**D**) DE ERV consistency between 25 pairs and all tumors vs. all normal samples of COAD. The red dots are ERVs upregulated and the green dots are ERVs downregulated in tumors.

In the TCGA COAD dataset, we used 25 available pairs of tumor and adjacent normal samples to compare ERV expression differences between cancer and normal colon samples. Among the 3220 ERVs, 2790 had non-zero expression across all samples and were used for further analyses. A principal components analysis (PCA) plot showed that the tumor samples were clearly separated from normal samples (Figure 2C). Comparing the mean ERV expression between tumor and normal samples showed that more ERVs were more highly expressed in the tumors (Figure 2D, the red curve for tumors shifted to the right). Unsurprisingly, differential expression analysis showed that 688 ERVs were elevated and 258 were depressed in the tumor samples (at FDR < 0.05 and absolute log2 fold change greater than 1, Figure 2E). These differentially expressed ERVs were mostly located in intergenic regions, followed by intronic regions of lncRNAs and protein coding genes (Figure 3A). Pathway enrichment analysis using ERV-associated genes (either overlapping or closest to known genes) showed significant enrichment of viral protein interaction with cytokine, cytokine receptor, and seleno-compound metabolism pathways for ERVs that were up expressed in tumors (Figure 3B), while ERVs that were down-expressed were enriched in vitamin, ascorbate, and aldarate metabolism and complement and coagulation cascades (Figure 3C).

To verify if the 25 tumor/normal pairs were representative in detecting differentially expressed ERVs between cancer and normal tissues, we also ran all available tumors compared to normal samples (305 vs. 41) and found that 810 out of 946 (86%) differentially expressed ERVs (DE-ERVs) from the paired samples were also significant DE-ERVs in the whole data analysis. The magnitude and direction of the changes were in high agreement (Figure 3D). Consistent with cell line data, our profiling in tissue samples also found that ERVs are expressed more in cancer tissues than their matching normal tissues.

### 3.4. ERV Expression-Defined “Subtypes” of COAD Are Associated with Immunogenic Scores and Tumor Mutation Burden

**Figure 4:**
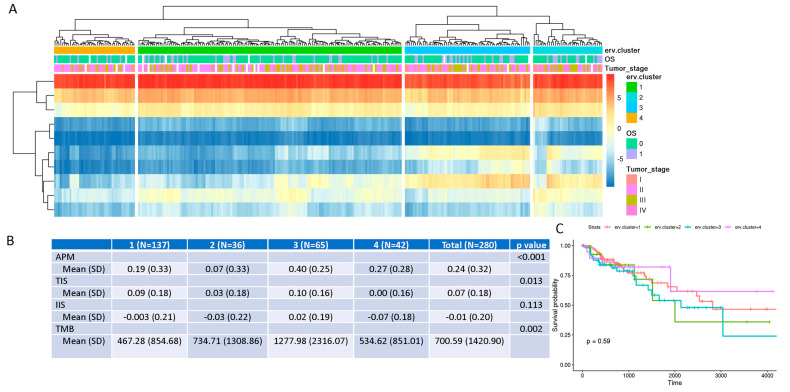
ERV expression-defined “subtypes” and their association with other clinical or molecular phenotypes. (**A**) Unsupervised clustering shows 4 distinct clusters. (**B**) Immunogenic score and tumor mutation burden association. (**C**) Kaplan–Meier survival curve for 4 different clusters.

Unsupervised clustering by ERV expression (ERVs from autosome chromosomes only for potential sex-related gene bias) on all tumors showed four distinct clusters (Figure 4A) and they were significantly associated with APM expression, TIS, and TMB (Figure 4B). These immunogenic scores were particularly higher in the samples of Cluster 3, although the ERV-defined clusters were not significantly associated with the DSS of the patients (*p* value 0.59, Figure 4C). The insignificant association may be caused by small the sample size and the fact that targeted immunotherapy was not available for the patients at that time. We also compared the differential protein gene expression of Cluster 3 with the other clusters and found 229 upregulated and 1193 downregulated genes (adjusted *p*-value less than 0.05 and absolute value of log2 fold change greater than 1). While no significant KEGG pathways were enriched for the upregulated genes, the top enriched GO biological process (adjusted *p* value < 0.05) was “Antimicrobial Humoral Response”. The downregulated genes were enriched in “neuroactive ligand–receptor interaction” and “ascorbate and aldarate metabolism” of KEGG pathways. In short, we identified an ERV expression-defined tumor cluster with high immunogenetic activity, which might benefit from immunotherapy.

### 3.5. Individual ERVs Associated with Disease Specific Survival

**Figure 5:**
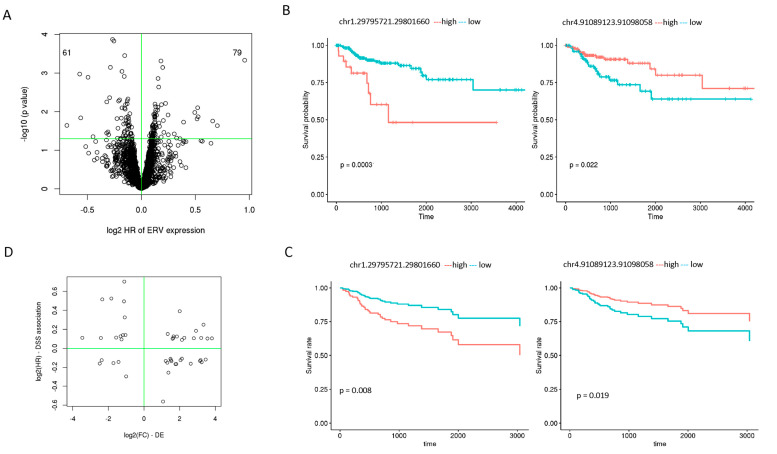
Disease-specific survival (DSS)-associated ERVs. (**A**) Volcano plot of log2 hazard ratio vs. log10 *p* value. The horizontal line is *p* value at 0.05. The numbers 61 and 79 on the left and right upper corner are the number of ERVs negatively and positively associated with patient survival, respectively. (**B**) Kaplan–Meier survival curve for 2 ERVs by univariate analysis. (**C**) Kaplan–Meier survival curve for 2 ERVs with adjustments for patient age, sex, and tumor stage. (**D**) ERVs that are both differentially expressed and associated with DDS.

With adjusting for patient age at diagnosis, sex, and tumor stage, we found 140 ERVs that were significantly associated with DSS at a *p* value less than 0.05 (the smallest *p* value was at 1 × 10^−4^, but none of the 140 passed the multiple testing adjusted q value less than 0.05), where higher expression of 79 was associated with poor patient survival and 61 with better survival (Figure 5A). The Kaplan–Meier survival curves of two ERVs (chr1.29795721.29801660 and chr4.91089123.91098058) with and without covariate adjustment are illustrated in Figure 5B (unadjusted) and C (adjusted). Among these DSS ERVs, 48 were also differentially expressed between tumors and normal samples, of which 15 had increased expression and worse survival and 16 had increased expression and better survival (Table 1).

## 4. Discussion

In this study we have profiled ERVs (manually curated) for their differential expression and associations with clinical and other molecular features. Compared to their paired normal tissues, most ERVs in CRC samples had increased expression, consistent with the notion that many ERVs in cancers may be reactivated as reported in lung squamous cell carcinoma [13]. This appears not to be in agreement with other reports where up- or down-expressed ERVs are about the same as in colon cancer [4] and lung adenocarcinoma [13]. The discrepancies are likely caused by different ERV annotations or analytical approaches, or the deregulation of ERVs may be cancer-type-specific. As an example, a previous study using the same TCGA COAD samples found 283 up- and 292 down-expressed ERVs from 3586 expressed ERVs [4]. The key differences from our study are that (1) they used the annotation consisting of 14,968 ERVs which are mostly digitally curated based on RepeatMasker and the RepBase database, and (2) their analysis used multiple mapped reads while ours used uniquely mapped reads only. Our study also found that ERV expression-defined subtypes were associated with immunogenic score or tumor mutation burden, which also supports the theory that they are immunogenic and can be used for immunotherapy by stimulating their expression [14]. We also identified ERVs that were associated with patient disease-free survival although their prognostic value needs to be further validated.

Although our findings are interesting, the biological interpretation is challenging as we know little about ERVs. The common approach is to use the ERV’s host (if an EVR is within another gene) or nearly coding gene as a surrogate. For the two ERVs whose expression is associated with patient survival, ERV chr1.29795721.29801660 is located in an intergenic region with reported 3C interaction and regulatory element. The closest gene to this ERV is ENSG00000284676, a novel lncRNA; however, no annotation or publication is available for this lncRNA. ERV chr4.91089123.91098058 is within an intron of CCSER1. CCSER1, along with other genes like FHIT, was found to play a significant role in genome stability and the cell division of colon cancer [15].

In this study we used uniquely mapped reads to ERVs to quantify their expression. Reads that span the regions or split reads were excluded to avoid counting reads from splicing transcripts, which other studies likely included. Excluding multiple mapped reads may underestimate the ERV expression or reduce ERV detection sensitivity with higher specificity; however, correctly assigning multiple mapped reads to each locus is a challenging problem. To address this challenge, the software tool “telescope” (https://github.com/mlbendall/telescope, accessed on 1 March 2025) was developed to assign the ambiguously mapped fragments to the most probable source transcript using a Bayesian statistical model [16]. We tested the tool on the CRC samples from the RNA atlas and found that the correlation between the telescope and our quantification was quite high (squared correlation coefficient R^2^ > 0.97) on the same ERV loci, suggesting that including multiple mapped reads does not have a big impact on the results, particularly for differential or relative expression analysis.

Our study has several limitations. Colon cancer risk factors such as smoking and alcohol likely affect ERV expression, but they are not easy to evaluate. We indeed performed association analysis between overall tumor ERV expression and available patient age and race and found no significant correlation (*p* value 0.17 and 0.12, respectively). Our findings are limited to digital data and lack laboratory or functional validation of their mechanisms. As a biomarker discovery lab, our primary focus is to identify useful and clinically testable markers that can be used for diagnosis or prognosis. One easily accessible medium is blood sampling. Currently we are conducting sample collection and testing if the reactivated ERVs can be tested from the plasma of colon cancer patients. Finally, we only profiled colon cancer tissue samples, and whether other cancers have similar changes remains to be seen. We plan to expand our studies to many other cancers.

## Figures and Tables

**Table 1:** Differentially expressed ERVs that are also associated with DSS.

ERV	DSS HR	Surv Pval	DE log2FC	DE Pval	DE Padj	DE Direction
chr14:73702885–73716167	1.63	2.31 × 10^−2^	−1.11	3.18 × 10^−7^	4.82 × 10^−6^	Down
chr19:56494761–56501703	1.44	1.36 × 10^−2^	−1.83	1.79 × 10^−11^	8.44 × 10^−10^	Down
chr17:43872266–43876352	1.43	1.55 × 10^−2^	−2.34	1.00 × 10^−15^	1.08 × 10^−13^	Down
chr19:38935163–38941835	1.41	1.05 × 10^−2^	−1.12	6.47 × 10^−6^	6.43 × 10^−5^	Down
chr12:76291617–76300850	1.31	1.87 × 10^−2^	2.00	1.82 × 10^−6^	2.12 × 10^−5^	Up
chr19:36323747–36332422	1.25	4.85 × 10^−2^	−1.08	1.16 × 10^−6^	1.49 × 10^−5^	Down
chr16:86739328–86747655	1.19	3.98 × 10^−2^	3.34	2.06 × 10^−17^	3.59 × 10^−15^	Up
chr6:144923391–144932448	1.14	4.54 × 10^−2^	2.92	8.27 × 10^−11^	3.30 × 10^−9^	Up
chr7:4590929–4600359	1.11	9.14 × 10^−3^	1.33	3.19 × 10^−3^	1.16 × 10^−2^	Up
chr15:51357586–51367580	1.10	2.55 × 10^−2^	−1.04	1.01 × 10^−2^	3.01 × 10^−2^	Down
chr2:64886311–64899831	1.10	2.55 × 10^−2^	−1.18	1.30 × 10^−3^	5.55 × 10^−3^	Down
chr4:179720511–179728321	1.09	1.92 × 10^−2^	1.84	7.08 × 10^−4^	3.36 × 10^−3^	Up
chr6:118574055–118578291	1.09	6.39 × 10^−3^	−1.31	4.95 × 10^−4^	2.49 × 10^−3^	Down
chr8:12037971–12041523	1.09	1.60 × 10^−2^	1.66	4.30 × 10^−3^	1.47 × 10^−2^	Up
chr1:247898555–247905892	1.08	2.17 × 10^−2^	−2.42	6.05 × 10^−6^	6.12 × 10^−5^	Down
chr11:23882492–23892875	1.08	3.05 × 10^−2^	2.80	7.56 × 10^−6^	7.30 × 10^−5^	Up
chr12:38123075–38131103	1.08	3.44 × 10^−2^	3.21	1.14 × 10^−6^	1.47 × 10^−5^	Up
chr19:54894290–54900597	1.08	2.55 × 10^−2^	2.26	3.24 × 10^−4^	1.74 × 10^−3^	Up
chr21:38224030–38229830	1.08	4.06 × 10^−2^	4.42	3.08 × 10^−10^	1.06 × 10^−8^	Up
chr4:4130683–4133908	1.08	1.19 × 10^−2^	−1.56	3.73 × 10^−3^	1.32 × 10^−2^	Down
chr6:123556843–123562657	1.08	2.29 × 10^−2^	−3.46	4.42 × 10^−12^	2.46 × 10^−10^	Down
chr6:123582329–123588017	1.08	3.02 × 10^−2^	1.65	7.54 × 10^−3^	2.36 × 10^−2^	Up
chr4:11653551–11659272	1.07	3.66 × 10^−2^	3.81	1.21 × 10^−7^	2.05 × 10^−6^	Up
chr8:11931841–11936717	1.07	2.34 × 10^−2^	1.60	1.30 × 10^−3^	5.55 × 10^−3^	Up
chrX:77077094–77086082	1.07	4.51 × 10^−2^	−1.26	5.94 × 10^−3^	1.95 × 10^−2^	Down
chrY:8994259–9004253	1.07	4.17 × 10^−2^	3.56	1.29 × 10^−5^	1.14 × 10^−4^	Up
chr11:89381303–89387559	1.06	3.61 × 10^−2^	2.13	4.48 × 10^−4^	2.26 × 10^−3^	Up
chr12:4018995–4025863	0.93	1.13 × 10^−2^	2.13	3.79 × 10^−5^	2.87 × 10^−4^	Up
chr8:60493460–60499925	0.93	2.21 × 10^−2^	1.48	1.84 × 10^−3^	7.30 × 10^−3^	Up
chr18:51112855–51117176	0.92	2.42 × 10^−2^	3.46	5.00 × 10^−8^	9.42 × 10^−7^	Up
chr4:23722376–23727827	0.92	2.25 × 10^−2^	−2.38	1.00 × 10^−9^	3.07 × 10^−8^	Down
chr5:34461058–34468430	0.92	1.89 × 10^−2^	2.05	1.34 × 10^−6^	1.65 × 10^−5^	Up
chrX:151550923–151562615	0.92	1.34 × 10^−2^	3.17	1.32 × 10^−8^	2.91 × 10^−7^	Up
chr8:7402289–7408615	0.92	3.54 × 10^−2^	1.51	1.36 × 10^−2^	3.84 × 10^−2^	Up
chr20:25394210–25403221	0.91	6.26 × 10^−3^	1.22	8.64 × 10^−3^	2.66 × 10^−2^	Up
chr6:137717275–137724946	0.91	5.27 × 10^−3^	3.14	1.20 × 10^−10^	4.65 × 10^−9^	Up
chr14:103234947–103242530	0.91	2.92 × 10^−2^	1.54	6.25 × 10^−5^	4.41 × 10^−4^	Up
chrX:97841727–97849389	0.91	1.71 × 10^−2^	−1.43	1.78 × 10^−3^	7.10 × 10^−3^	Down
chr9:64870612–64878934	0.90	1.98 × 10^−2^	3.24	1.45 × 10^−9^	4.26 × 10^−8^	Up
chr11:58996950–59006462	0.90	8.61 × 10^−3^	1.31	5.78 × 10^−4^	2.82 × 10^−3^	Up
chr4:115950630–115956482	0.90	3.20 × 10^−2^	−1.72	2.70 × 10^−3^	1.01 × 10^−2^	Down
chr6:63321317–63335844	0.90	1.23 × 10^−3^	−2.48	5.14 × 10^−10^	1.67 × 10^−8^	Down
chr4:77630582–77637015	0.90	3.47 × 10^−2^	2.62	1.47 × 10^−9^	4.28 × 10^−8^	Up
chr4:58458021–58466147	0.89	2.10 × 10^−2^	1.81	3.38 × 10^−3^	1.22 × 10^−2^	Up
chr9:29775512–29780684	0.89	4.75 × 10^−2^	1.77	2.88 × 10^−3^	1.06 × 10^−2^	Up
chr22:30676787–30679926	0.84	1.49 × 10^−4^	1.37	4.00 × 10^−6^	4.26 × 10^−5^	Up
chr3:11247257–11254838	0.82	4.36 × 10^−3^	−1.00	4.07 × 10^−5^	3.03 × 10^−4^	Down
chr11:17370179–17379293	0.68	1.46 × 10^−2^	1.07	7.14 × 10^−6^	6.97 × 10^−5^	Up

## Data Availability

The raw data presented in this study are openly available in GEO (accession number: GSE138734) and GDC data portal (TCGA project) for colon cancer at https://portal.gdc.cancer.gov/. The raw sequence data in TCGA has controlled access and users need to apply for approval for its usage.

## Supplementary Materials

The following supporting information can be downloaded at: https://www.mdpi.com/article/10.3390/genes16080988/s1, Figure S1: Pair-wise correlation for each colon cell line between polyA and totalRNA protocols; Figure S2: ERV expression in other cancer cell lines and normal cells (breast, lung and prostate); Figure S3: ERV expression in 7 cancer and 1 normal colon cells.

## Abbreviations

The following abbreviations are used in this manuscript:

ERVs	Human endogenous retroviruses
COAD/CRC	Colon adenocarcinoma/colorectal carcinoma
TCGA	The Cancer Genome Atlas
DSS	Disease-specific survival
GEO	Gene expression omnibus
IIS	Immune infiltration score
APM	Antigen processing and presenting machinery
TMB	Tumor mutation burden
RPKM	Reads per kilobase of transcript per million mapped reads
GO	Gene ontology
KEGG	Kyoto Encyclopedia of Genes and Genomes
