# Supplementary material for: Abnormal ERV Expression and Its Clinical Relevance in Colon Cancer
Source: Genes (Basel). 2025 Aug 21;16(8):988. doi: 10.3390/genes16080988 (PMC12385626; doi:10.3390/genes16080988)
Supplement: Supplementary file 1 [file genes-16-00988-s001.zip › genes-3757981-supplementary.pdf]

Abnormal ERV expression and its clinical relevance in colon cancer – supplementary materials

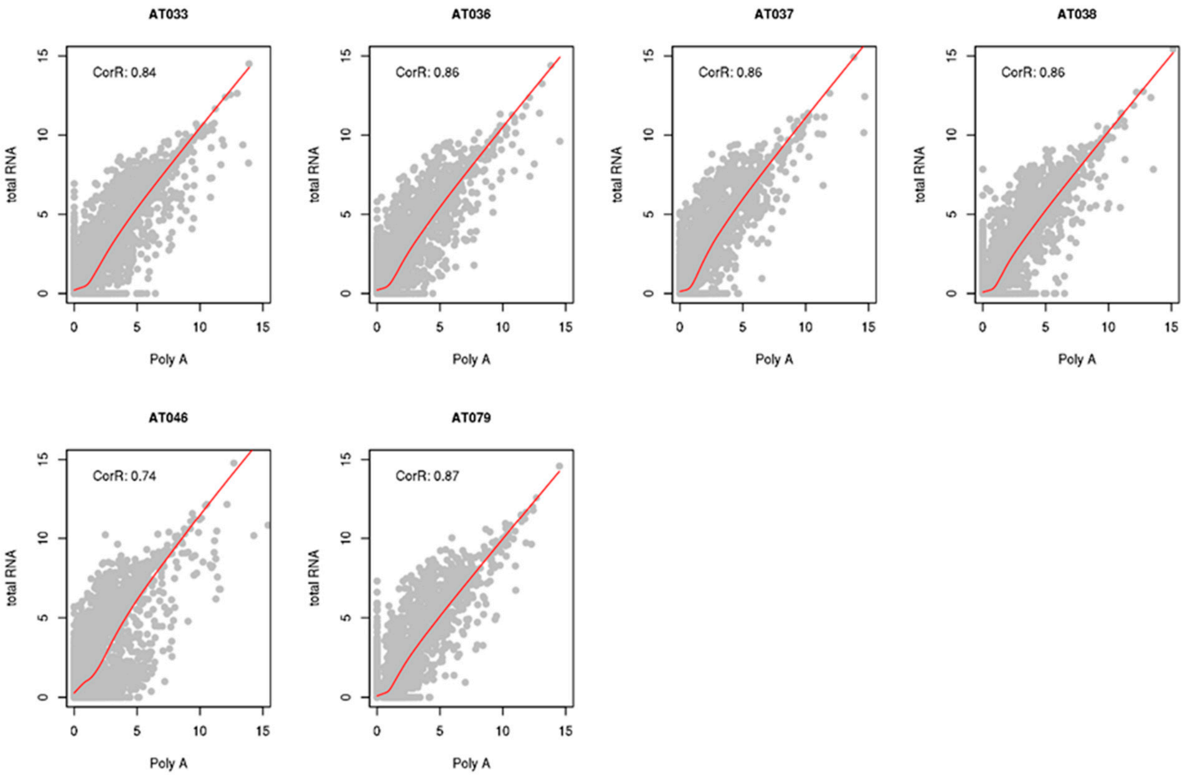

Figure S1: Pair-wise correlation for each colon cell line between polyA and totalRNA protocols. The correlation coefficient is generally high (around 0.85) except one cell (0.74). Most ERVs are quantified at higher level in totalRNA protocol compared to polyA protocol.

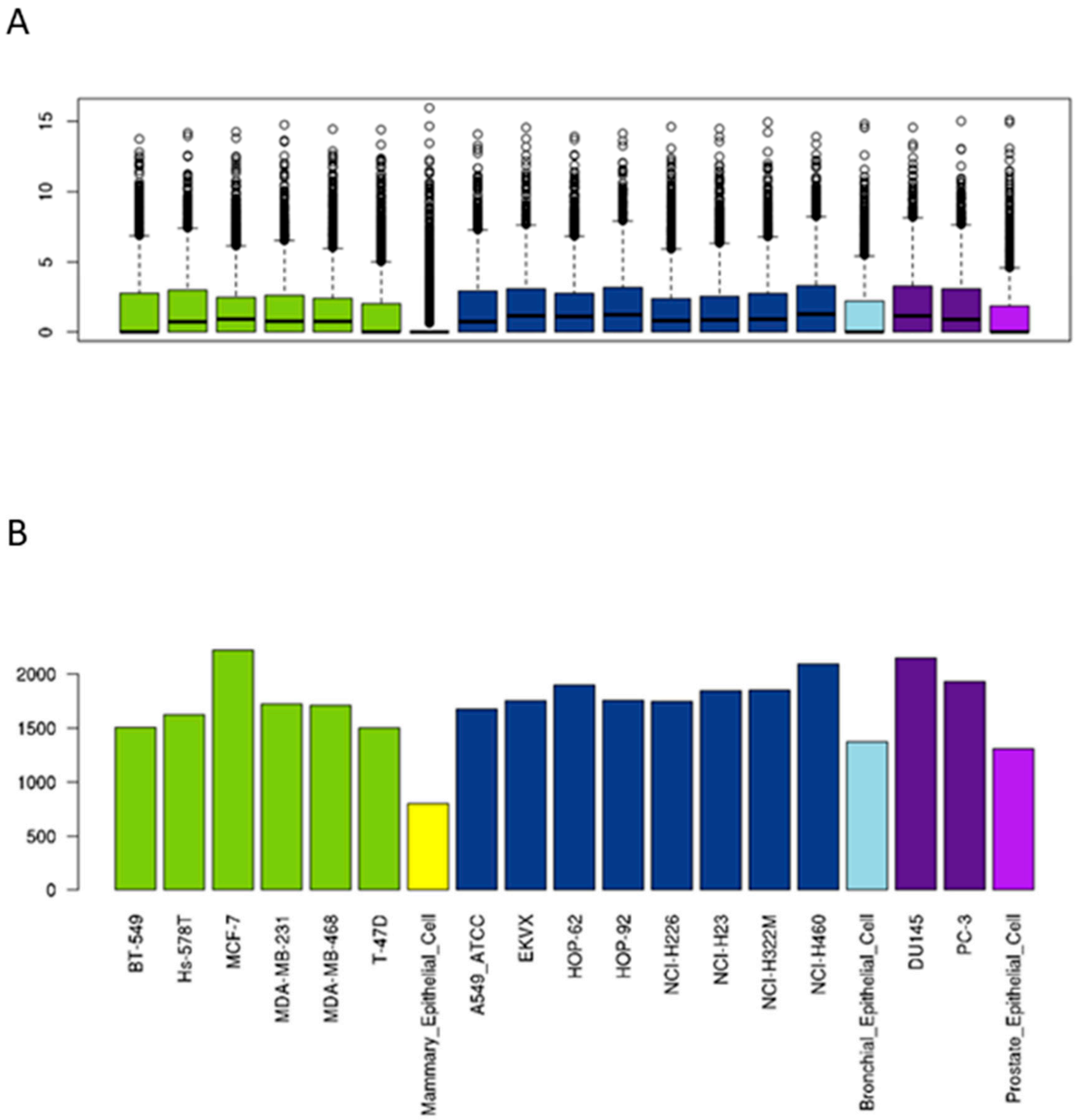

Figure S2: ERV expression in other cancer cell lines and normal cells (breast, lung and prostate). A. Boxplot for range of ERV expression in each cell line where a horizontal bar within a box is the median expression. Cell lines are ordered by breast (green), lung (blue) and prostate (purple) from left to right. The matching normal cell line has light color at the end of each cancer type. B. the number of ERVs detected in each cell line.

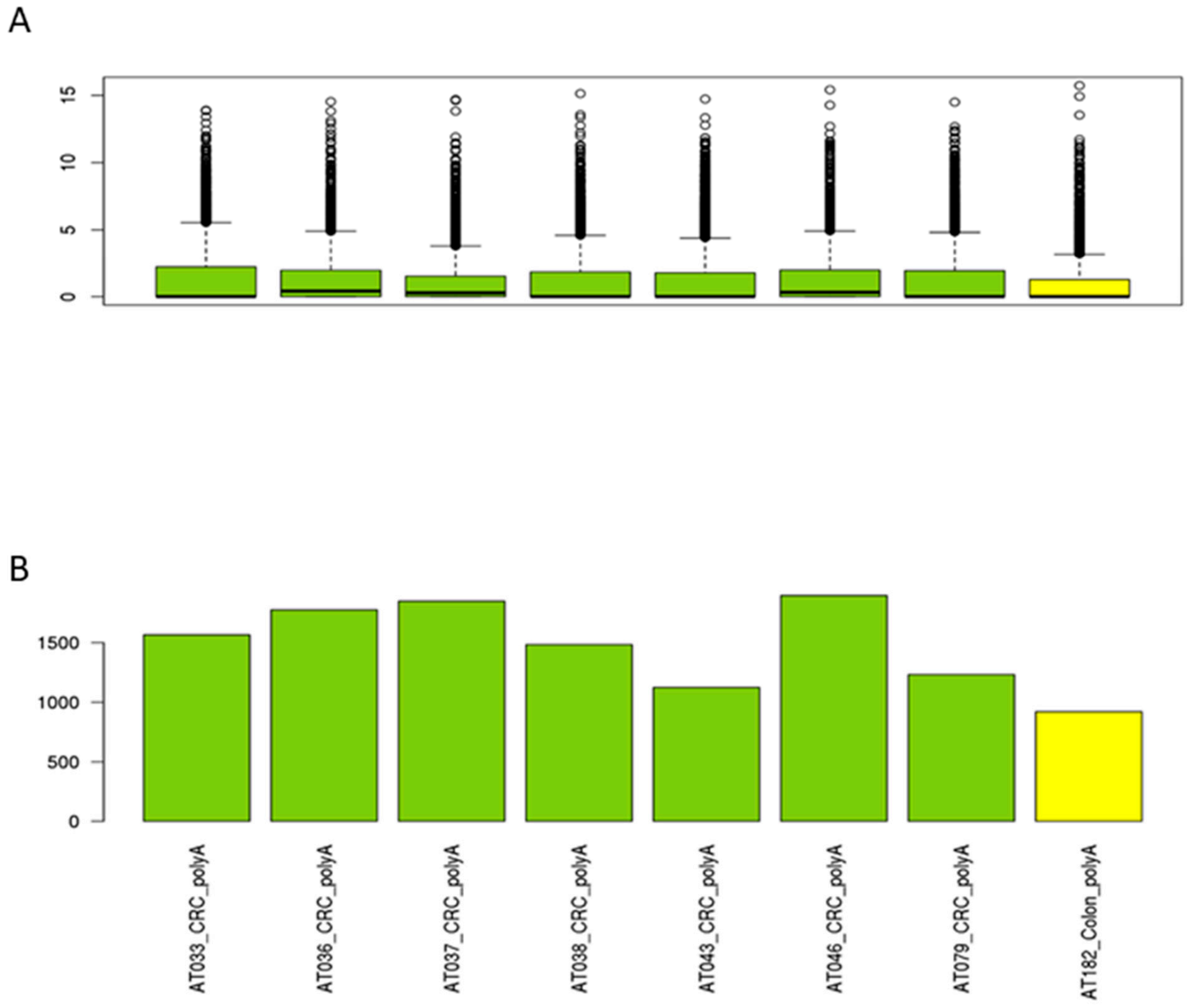

Figure S3: ERV expression in 7 cancer (green) and 1 normal colon cells (yellow). A. Boxplot for range of ERV expression in each cell line where a horizontal bar within a box is the median expression. B. the number of ERVs detected in each cell line.
